# N-glycan alterations are associated with drug resistance in human hepatocellular carcinoma

**DOI:** 10.1186/1476-4598-6-32

**Published:** 2007-05-09

**Authors:** Takeaki Kudo, Hiroaki Nakagawa, Masato Takahashi, Jun Hamaguchi, Naoya Kamiyama, Hideki Yokoo, Kazuaki Nakanishi, Takahito Nakagawa, Toshiya Kamiyama, Kisaburo Deguchi, Shin-Ichiro Nishimura, Satoru Todo

**Affiliations:** 1 Department of General Surgery, Graduate School of Medicine, Hokkaido University, Japan; 2 Graduate School of Advanced Life Science, Hokkaido University, Japan; 3 Department of Sensory Physiology, Asahikawa Medical College, Japan

## Abstract

**Background:**

Correlations of disease phenotypes with glycosylation changes have been analysed intensively in the tumor biology field. Glycoforms potentially associated with carcinogenesis, tumor progression and cancer metastasis have been identified. In cancer therapy, drug resistance is a severe problem, reducing therapeutic effect of drugs and adding to patient suffering. Although multiple mechanisms likely underlie resistance of cancer cells to anticancer drugs, including overexpression of transporters, the relationship of glycans to drug resistance is not well understood.

**Results:**

We established epirubicin (EPI) – and mitoxantrone (MIT) – resistant cell lines (HLE-EPI and HLE-MIT) from the human hepatocellular carcinoma cell line (HLE). HLE-EPI and HLE-MIT overexpressed transporters MDR1/ABCB1 and BCRP/ABCG2, respectively. Here we compared the glycomics of HLE-EPI and HLE-MIT cells with the parental HLE line. Core fucosylated triantennary oligosaccharides were increased in the two resistant lines. We investigated mRNA levels of glycosyltransferases synthesizing this oligosaccharide, namely, N-acetylglucosaminyltransferase (GnT)-IVa, GnT-IVb and α1,6-fucosyltransferase (α1,6-FucT), and found that α1,6-FucT was particularly overexpressed in HLE-MIT cells. In HLE-EPI cells, GnT-IVa expression was decreased, while GnT-IVb was increased. Both GnT-IVs were downregulated in HLE-MIT cells. HLE-MIT cells also showed decreases in fucosylated tetraantennary oligosaccharide, the product of GnT-V. GnT-V expression was decreased in both lines, but particularly so in HLE-MIT cells. Thus both N-glycan and glycosyltransferase expression was altered as cells acquired tolerance, suggesting novel mechanisms of drug resistance.

**Conclusion:**

N-glycan and glycosyltransferase expression in HLE-EPI and HLE-MIT were analysed and presented that glycans altered according with acquired tolerance. These results suggested novel mechanisms of drug resistance.

## Background

Several chemotherapeutic agents are used to treat malignant tumors. However, acquired resistance to these agents frequently occurs and is a serious problem during treatment. One cause of resistance is elevated expression or activity of ATP-binding cassette (ABC) transporters, such as multidrug resistant protein 1 (MDR1/ABCB1) and breast cancer resistance protein (BCRP/ABCG2). Both transporters are membrane glycoproteins containing N-glycans.

Oligosaccharide expression is highly relevant to many cancers. Many studies show that alterations in N-linked oligosaccharides of tumor cells are associated with carcinogenesis, invasion and metastasis [1,2]. Yamashita et al reported that products of N-acetylglucosaminyltransferase (GnT)-IV, GnT-V and α1,6-fucosyltransferase (α1,6-FucT) are all increased in hepatocellular carcinoma [3]. However the relationship between chemoresistance and N-glycans has been investigated in very few studies.

Asparagine (Asn) 596 of BCRP is normally glycosylated. Studies in which this residue was mutated indicate that N-glycosylation at Asn 596 is not essential for BCRP expression, trafficking to the plasma membrane, or function [4,5]. On the other hand, a multi-drug-resistant cell line in which MDR1 is highly expressed showed reduced tolerance following loss of MDR1 function and decreased glycosylation mediated by tunicamycin, which inhibits biosynthesis of glycan precursors [6].

A different study showed that the activity of β-galactoside α2,6-sialyltransferase I in human colon cancer cells was lost when they acquired methotrexate resistance and that glycan structures on the cell membrane were altered [7]. N-linked glycans on α5β1 integrin in cisplatin-resistant head and neck cancer cell showed reduced β1,6-N-acetylglucosamine branches compared to the parent line [8]. These studies have detected alterations in N-glycan with lectins, but little information is still available on precise structural alteration of N-glycans in drug-resistant cells.

Epirubicin (EPI) and mitoxantrone (MIT) are widely used for cancer chemotherapy. We have established EPI-resistant cells (HLE-EPI), which overexpress MDR1, and MIT-resistant cells (HLE-MIT), which overexpress BCRP, from HLE human hepatocellular carcinoma cells [9]. To detect N-glycan structures that potentially underlie anticancer drug resistance, we compared the glycomics of HLE, HLE-EPI, and HLE-MIT lines. We also compared expression of mRNA encoding glycosyltransferases involved in the synthesis of glycans potentially mediating resistance in these lines.

## Results

### Oligosaccharides detailed structures and ratios

**Figure 1:**
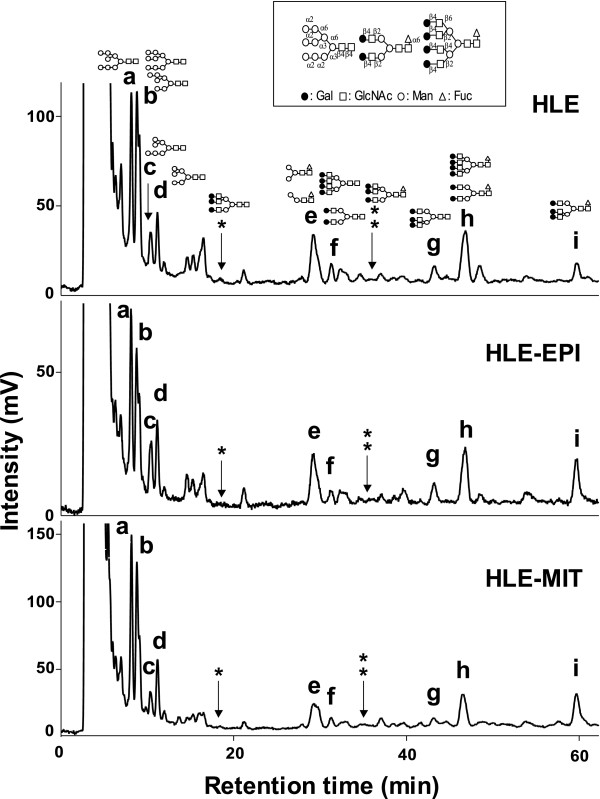
**Chromatograms of PA-*N*-glycans identified on ODS columns**. Letters and asterisks over peaks correspond with Table 1.

**Figure 2:**
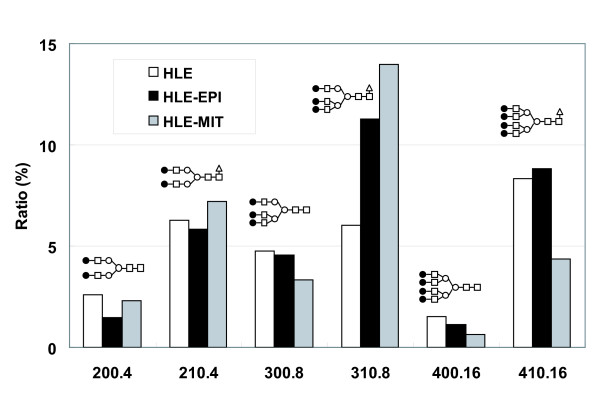
**Alterations of complex type oligosaccharide ratios in cell lines**. Ratios were calculated based on amide column isolation following separation using ODS columns.

Pyridylaminated (PA)-oligosaccharide samples of HLE, HLE-EPI and HLE-MIT were separated and their elution positions were analyzed on octadecylsilyl (ODS) columns (Fig. 1). Separated sample peaks were analyzed on amide columns, and structures of oligosaccharides contained in each peak were determined using 2-dimensional mapping (2-DM) strategies. Molar ratios of oligosaccharides were calculated based on peak areas from the ODS analysis (Table 1). Quantitative analysis of 9 peaks on the ODS column showed that the level of one complex type oligosaccharide [Galβ1,4-GlcNAcβ1,2-Manα1,6-(Galβ1,4-GlcNAcβ1,4-(Galβ1,4-GlcNAcβ1,2-)Manα1,3-)Manβ1,4-GlcNAcβ1,4-(Fucα1,6-)GlcNAc (code No: 310.8)], a triantennary core-fucosylated oligosaccharide structure, increased in drug-resistant cells, particularly HLE-MIT. We then analyzed more detail ratios of complex type oligosaccharides using further amide analysis (Fig. 2). Among those oligosaccharides, only 310.8 increased in drug-resistant cells. Levels of non-fucosylated triantennary oligosaccharides (300.8) and biantennary oligosaccharides (200.4 and 210.4) were unchanged in resistant cells, but tetraantennary glycans (400.16 and 410.16) decreased in HLE-MIT.

**Table 1:** *N*-Glycan ratios from HLE, HLE-EPI and HLE-MIT cells analyzed on ODS columns

	Oligosaccharide	Molar ratio(%)		
		
		HLE	HLE-EPI	HLE-MIT
a	M8.1	15.06	16.12	18.48
b	M7.2 + M9.1	26.83	21.02	24.77
c	M7.1	5.63	7.88	3.95
d	M6.1	6.45	7.80	8.29
e	010.0 + 010.1	16.56	14.07	12.70
f	200.4 + 400.16	4.11	2.59	2.91
g	300.8	4.77	4.57	3.34
h	210.4 + 410.16	14.57	14.67	11.58
i	310.8	6.01	11.29	13.97
*	300.18	Detected (< 1.00)		
* *	310.18	Detected (<1.00)		

Detailed structures are shown in Fig. 1 and code numbers of oligosaccharides follow the designations of Takahashi [36, 37].

### Expression of glycosyltransferases

**Figure 3:**
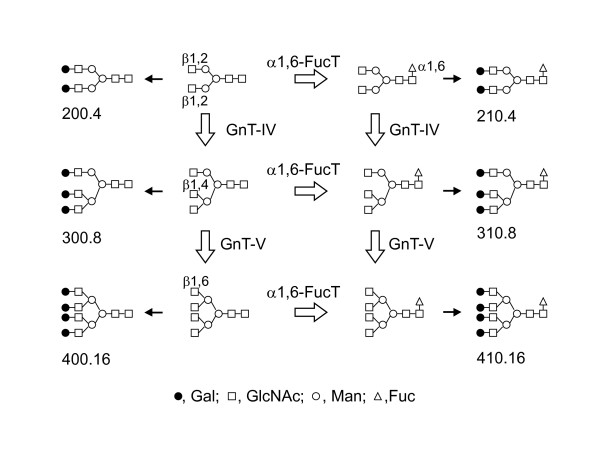
**Biosynthesis pathway of complex type N-glycans**. The reactions of α1,6-FucT, GnT-IV and GnT-V and complex type N-glycan structures in this study are highlighted. GnT-IV and GnT-V increase number of antenna, and some of them are modified by α1,6-FucT. Then, they are galactosylated by β1,4- galactosyltransferase as shown as black arrows. The products of GnT-V were modified by GnT-IV in this study, so we have indicated that GnT-V works after GnT-IV in the biosynthetic pathway. However, it is possible that GnT-V may act at an earlier step than GnT-IV.

**Figure 4:**
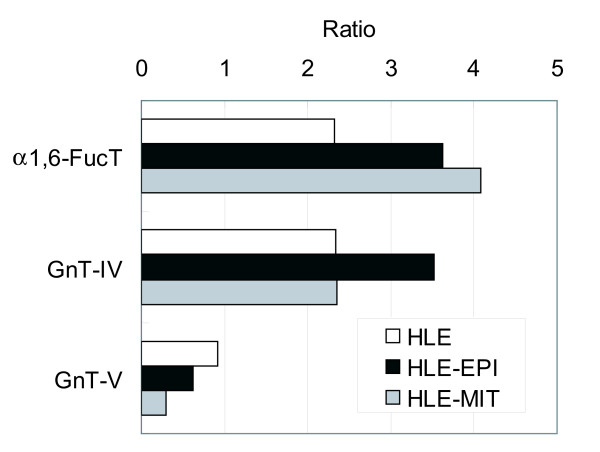
**Glycosyltransferase activities estimated by ratios of *N*-glycans**. Alpha1,6-FucT, (210.4+310.8+410.16)/(200.4+300.8+400.16); GnT-IV, (300.8+310.8+400.16+410.16)/(200.4+210.4); and GnT-V, (400.16+410.16)/(300.8+310.8).

The biosynthetic pathway of N-glycans shown in Figure 3 highlights importance of enzymes α1,6-FucT, GnT-IV and GnT-V. We estimated the enzymatic activity of α1,6-FucT from the ratio of core-fucosylated (210.4, 310.8, 410.16) to non-fucosylated (200.4, 300.8, 400.16) oligosaccharides, GnT-IV from triantennary plus tetraantennary (300.8, 310.8, 400.16, 410.16)/biantennary (200.4, 210.4) oligosaccharides, and GnT-V from tetraantennary (400.16, 410.16)/triantennary oligosaccharides (300.8, 310.8) (Fig 4). Estimated activities of α1,6-FucT and GnT-IV were increased in drug resistant cells, while GnT-V was decreased.

**Figure 5:**
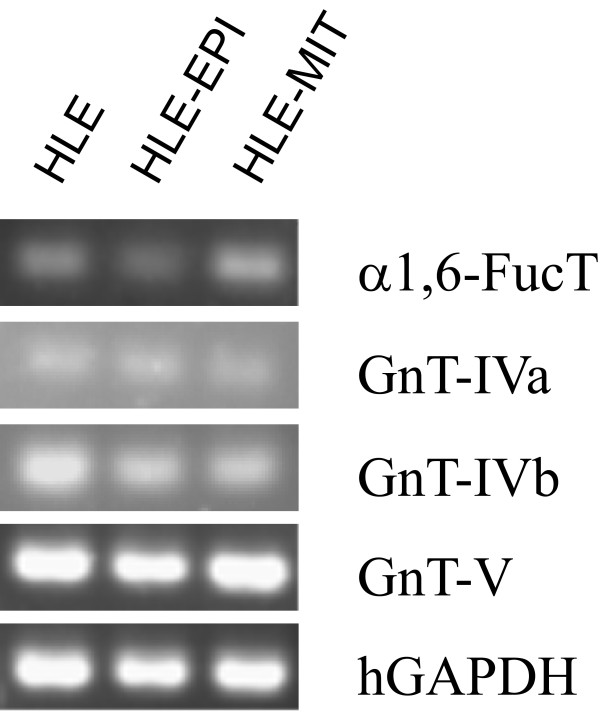
**RT-PCR analysis of glycosyltransferase mRNA expression in each cell line**. Detailed experimental procedures are in Materials and Methods. Human glyceraldehyde-3-phosphate dehydrogenase (hGAPDH) is reported for internal controls.

To analyze expression of these glycosyltransferases, RT-PCR was performed (Fig 5). α1,6-FucT in HLE-EPI levels were slightly decreased, and GnT-IVa in drug-resistant cells was slightly increased; however, the difference between parental and drug-resistant cells remained unclear. We performed real-time RT-PCR analysis to confirm these results more quantitatively (Table 2). α1,6-FucT expression was increased in drug-resistant cells, especially in HLE-MIT. GnT-IVa was decreased in drug-resistant cells, but GnT-IVb was increased in HLE-EPI and decreased in HLE-MIT lines. GnT-V was decreased in drug-resistant cells.

**Table 2:** Glycosyltransferase gene expression analyzed quantitatively by real-time RT-PCR

**Enzyme**	**Cell line**	**mRNA expression (ratio against HLE)**	**S.D**.	**p Value (vs HLE)**
**α1,6-FucT**	HLE	1.000	0.212	-
	HLE-EPI	1.100	0.062	0.402
	HLE-MIT	1.832	0.454	0.016

**GnT-IVa**	HLE	1.000	0.136	-
	HLE-EPI	0.037	0.005	7.897 × 10^-6^
	HLE-MIT	0.003	0.001	6.421 × 10^-6^

**GnT-IVb**	HLE	1.000	0.170	-
	HLE-EPI	1.304	0.124	0.028
	HLE-MIT	0.561	0.085	0.004

**GnT-V**	HLE	1.000	0.428	-
	HLE-EPI	0.302	0.105	0.019
	HLE-MIT	0.122	0.044	0.006

mRNA expression is based on an average of 5 experiments. S.D.; standard deviation.

## Discussion

To elucidate the mechanism of drug resistance, we investigated N-glycans and glycosyltransferases in parental and drug-resistant cells. The structures of fifteen types of oligosaccharides were determined, and one in particular–310.8–was dramatically increased in resistant cells. This N-glycan is synthesized by GnT-IV and α1,6-FucT. We examined expression of these two glycosyltransferases together with GnT-V, which is reported to be associated with cancer malignancy.

Both core α1,6-fucosylated N-glycans and α1,6-FucT expression are associated with cancer. Fucosylation of serum proteins has been reported as tumor marker on IgG and α-fetoprotein in hepatocellular carcinoma [10,11] and haptoglobin in pancreatic cancer [12]. Our glycomic analysis showed high core-fucosylation in both resistant lines, and particularly high expression of α1,6-FucT in HLE-MIT. These results support the idea that fucosylation anomalies occur in cancer. Proteins such as IgG, α-fetoprotein and haptoglobin contain mainly biantennary N-glycans. Here, we observed that only triantennary–not bi- and tetraantennary–N-glycans were increased, although we cannot as yet account for why these changes occur. Alterations in core fucosylation are associated with changes in protein function: fucose removal from oligosaccharides of the scFv-Fc domain of human IgG1 results in significantly enhanced cellular cytotoxicity [13-15]. In vivo, Wang showed that α1,6-FucT null mice showed semi-lethality and severe growth retardation [16]. These mice also overexpressed matrix metalloproteinases-12 and -13, an alteration associated with pathological lung phenotypes including emphysema-like changes. Nakagawa reported that this core α1,6-Fuc works as a signal of secreting carrier protein to bile ducts in a liver [17]. This core α1,6-Fuc is also essential for epidermal growth factor function [18]. Thus, core α1,6-Fuc has critical functions, and proteins bearing 310.8 are presumed to protect cancer cells from anticancer drugs.

GnT-IV is a glycosyltransferase functioning in 310.8 synthesis. GnT-IV activity is associated with metastasis of colorectal carcinoma [19], is increased in choriocarcinoma [20], and is decreased in renal cell carcinoma [21]. Human chorionic gonadotropins from patients with choriocarcinoma contain oligosaccharides modified by GnT-IV [22] and Ide et al investigated the relationship between pancreatic cancer and GnT-IV [23]. From these results show that GnT-IV functions are not inherently good or bad in the context of cancer. Here, we show that GnT-IV products increase in both resistant cells, but expression of GnT-IVs were not correlated with products. Detailed oligosaccharide structural analysis and evaluation of glycosyltransferase expression were not precisely correlating, most likely because N-glycan biosynthesis depends on several variables, including carrier protein character and synthesis, a supply of sugar nucleotides, a precise enzyme location, and degradation of enzyme and products. Such a discrepancy suggests that both glycan profiles and expression or activation of glycosyltransferases must be defined.

GnT-V is reportedly an indicator of tumor malignancy [24,25]. However Nakahara et al reported that GnT-V activity was correlated with cisplatin sensitivity in squamous cell carcinoma [8], and Ishimura showed that GnT-V and its resultant β1,6-branched N-linked oligosaccharides were closely related to low malignant potential and good prognosis in bladder cancer patients [26]. In our study, expression of GnT-V and glycomic analysis of the tetraantennary (400.16, 410.16)/triantennary (300.8, 310.8) ratio suggested that GnT-V activity in drug-resistant cells was lower than in parental cells. These findings agree with Nakahara's and Ishimura's results that GnT-V activity is not simply associated with outcomes such as high grade malignancy, poor prognosis, or anticancer drug resistance. N-glycan structures from the 3 cell lines we examined were almost the same, which is natural because the 2 drug-resistant cell lines were established from the same parental cell line. However, there were some differences in N-glycan ratios and gene expression between HLE-EPI and HLE-MIT. EPI and MIT have similar structures and the same mechanism of cell toxicity: binding DNA and inhibiting topoisomerase II activity [27]. Nevertheless, EPI produces a hydroxyl radical but MIT does not [28], and Moriyama reported that gene expression patterns in response to EPI and MIT differed [27]. In addition, many mechanisms of drug resistance have been reported even against the same reagents, so the differences we saw between HLE-EPI and HLE-MIT are acceptable.

N-glycans bind proteins, and the combinations of glycan and protein may be important in their function [1,2]. Despite many genomic and proteomic studies of drug resistance [29,30], no dramatic change in the abundance of a protein has been clearly associated with drug resistance. It is more likely that the altered N-glycans we observed bind various proteins, and that altered N-glycans associated with several different proteins affect the drug-resistance mechanisms.

The ABC transporter is a key factor in drug resistance. Our cell lines overexpressed MDR1/ABCB1 (HLE-EPI) and BCRP/ABCG2 (HLE-MIT). Previously, correlation between N-glycan presence and transporter expression, localization, and function were investigated [4-6,31]. However, there is no information concerning potential direct effects on transporter activity by N-glycan structures, because these studies inhibited glycosylation either by mutating N-glycan-bearing asparagine residues or by tunicamycin treatment. Determination of N-glycan structures of transporters and whether transporter activity is altered by changes in those structures is a topic for further studies.

## Conclusion

Here, we analyzed glycomics of HLE, HLE-EPI, and HLE-MIT lines and detected quantitative changes in N-glycans, that core-fucosylated triantennary N-glycans are clearly increased in anticancer drug-resistant cells. Ours is the first study identifying precise oligosaccharide structures and related glycosyltransferase activities that may be involved in anticancer drug resistance.

## Methods

### Chemicals

Trypsin and sodium cyanoborohydride were purchased from Sigma-Aldrich Co. (St. Louis, MO), α-chymotrypsin and pronase were from Calbiochem Co. (Darmstadt, Germany), peptide-N-glycosidase F (PNGase F) was from Hoffman-La Roche (Basel, Switzerland), glucose oligomers (4 – 20) were from Seikagaku Co. (Tokyo, Japan), and 2-aminopyridine was from Wako Pure Chemicals (Osaka, Japan). Bio-Gel P-4 (200–400 mesh) was obtained from Bio-Rad Laboratories (Hercules, CA), Sephadex G-15 from Amersham Biosciences (Uppsala, Sweden), an octadecyl-bonded silica (ODS) column, ShimPack HRC-ODS, (6.0 mm i.d. × 150 mm) from Shimadzu Co. (Kyoto, Japan), and TSKgel Amide 80 column (Amide column) (4.6 mm i.d. × 250 mm) from Tosoh (Tokyo, Japan). EPI hydrochloride was obtained from Pfizer Japan Inc. (Tokyo, Japan). MIT hydrochloride was purchased from Sigma-Aldrich (St Louis, MO).

### Cell lines and culture

HLE human hepatocellular carcinoma cells were purchased from JCRB Cell Bank (Osaka, Japan). EPI-resistant (HLE-EPI) and MIT-resistant (HLE-MIT) cells were established from HLE as described [9]. Cells were cultured in Dulbecco's modified Eagle medium with 10 % (v/v) fetal bovine serum [32]. HLE-EPI or HLE-MIT cells were maintained by exposing them to the respective drugs at 32 ng/ml for two days before passage. Cells were plated onto 100 mm culture dishes at a density of 1 × 10^6 ^cells/dish. Twelve dishes per line were cultured simultaneously. Cells were harvested using EDTA and trypsin and pelleted by centrifugation.

### Preparation and derivatization of N-linked oligosaccharide

Cells from four dishes were pooled to make one sample, washed twice with phosphate buffered saline, resuspended in water, heated at 90°C for 15 min, and lyophilized. Dried cells (approximately 5 mg each) were digested with 50 μg each of trypsin and α-chymotrypsin, and N-linked oligosaccharides were released from peptides with 5 U of PNGase F. Peptides were then digested with 50 μg pronase. Each step was done in 10 mmol/l ammonium bicarbonate, pH 8.0, at 37°C overnight. Oligosaccharides were purified on Bio-gel P-4 columns and reductively aminated with 2-aminopyridine and sodium cyanoborohydride [33,34]. PA-oligosaccharides were purified by gel filtration on a Sephadex G-15 column (1.0 × 38 cm) with 10 mmol/l ammonium bicarbonate. To release sialic acids, oligosaccharides were heated 1 h at 90°C at pH 2.0 (with HCl).

### PA-oligosaccharide profiling by the 2-DM method using high performance liquid chromatography (HPLC)

PA-oligosaccharides were analyzed by the 2-DM method using a Hitachi L-7000 HPLC system (Hitachi High-Technologies Co., Tokyo, Japan) [35,36]. PA-oligosaccharide samples were first analyzed on an ODS column. Elution was performed at a flow rate of 1.0 ml per minute at 55°C using a gradient system. Solvent A was10 mmol/l sodium phosphate buffer (pH 3.8) and solvent B was 0.5% (v/v) 1-butanol added in solvent A. The column was equilibrated with solvent (A : B = 80 : 20 (v/v)), and then the injection ratio of solvent B was increased linearly to 50 % over 60 min. Eluted PA-oligosaccharides were detected with a fluorescence spectrometer, and excitation and emission wavelengths were 320 nm and 400 nm, respectively. Separated oligosaccharides of each peak of the ODS column were analyzed using an Amide column at a flow rate of 1.0 ml per minute at 40°C with solvent C (3% (v/v) acetic acid-triethylamine (pH 7.3)/acetonitrile 35 : 65) and solvent D (3% (v/v) acetic acid-triethylamine (pH 7.3)/acetonitrile 50 :50). The column was initially equilibrated with only solvent C, and elution was performed using a linear gradient to solvent (C:D = 40:60 (v/v)) in 30 min. PA-oligosaccharides were also monitored by fluorescence. The elution position of PA-oligosaccharide on each column was converted to glucose unit (GU) values to make reproducibility. The relative amount of PA-oligosaccharide was calculated based on the peak area analyzed by software used in conjunction with the HPLC system. Oligosaccharide structures were suggested by comparing elution positions with data reported in the same analytical conditions [36,37]. Code numbers of oligosaccharide structures described in this manuscript are according these references.

### Reverse transcription (RT)-polymerase chain reaction (PCR)

Total RNA was isolated from cultured cells with ISOGEN (Nippon Gene, Tokyo, Japan) according to the manufacturer's protocol. 5 μg total RNA was used with ReverTra Ace (Toyobo Co., Osaka, Japan) for reverse transcription. The reaction mixture was according to the manufacturer's protocol with 50 ng total RNA. Primer sequences were as follows: 5'-CAGACAGATGGAGCAGGTGA-3' (forward) and 5'-ACCACATGATGGAGCTGACA-3' (reverse) for α1,6-fucosyltransferase (fucosyltransferase 8, α1,6-FucT); 5'-ACCAAGGGCATACGCTGGAG-3' (forward) and 5'-GTTCTTGGTTGCCGCTATGGA-3' (reverse) for N-acetylglucosaminyltransferase (GnT)-IVa; 5'-ACTTCATCCGCTTCCGCTTC-3' (forward) and 5'-TCCTTGTCTGACTGAGGGTTGT-3' (reverse) for GnT-IVb; 5'-CTCAGCGCCCTACAGGTCAA-3' (forward) and 5'-CTTGATGAAGTCCCGGCAGG-3' (reverse) for GnT-V; and 5'-GCCTCCTGCACCACCTG-3' (forward) and 5'-CGACGCCTGCTTCACCACCTTCT-3' (reverse) for GAPDH. Product sizes of α1,6-FucT, GnT-IVa, GnT-IVb, GnT-V and GAPDH were 170 bp, 144 bp, 142 bp, 291 bp and 351 bp, respectively.

PCR was performed using the Expand High Fidelity PCR System (Roche, Basel, Switzerland). PCR cycles started with 2 min at 94°C and then 35 cycles of 15 sec at 94°C, 30 sec at 56°C, and 30 sec at 72°C for α1,6-FucT and GAPDH; 45 cycles of 15 sec at 94°C, 30 sec at 60°C and 30 sec at 72°C for GnT-IVa, GnT-IVb and GnT-V. PCR products were electrophoresed on 1.5% agarose gels and stained with ethidium bromide.

### mRNA quantification

Real-time RT-PCR was performed using a LightCycler™ (Roche) with a QuantiTect SYBR Green PCR Kit (QIAGEN K.K., Tokyo, Japan). Reaction mixtures were made according to the manufacturer's instruction with 50 ng total RNA. Primer sequences were the same as for RT-PCR. Real-time PCR cycles started with 15 min at 95°C and then 45 cycles of 15 sec at 94°C, 20 sec at 56°C and 20 sec at 72°C for α1,6-FucT; 50 cycles of 15 sec at 94°C, 20 sec at 60°C and 15 sec at 72°C for GnT-IVa and GnT-IVb; 50 cycles of 15 sec at 94°C, 20 sec at 60°C and 25 sec at 72°C for GnT-V; and 45 cycles of 15 sec at 94°C, 20 sec at 56°C and 20 sec at 72°C for GAPDH. Expression levels of α1,6-FucT, GnT-IVa, GnT-IVb, and GnT-V were expressed as ratios relative to GAPDH.

## Abbreviations

ABC, ATP-binding cassette; BCRP, breast cancer resistance protein; 2-DM, 2-dimensional mapping; EPI, epirubicin; Fuc, fucose; FucT, fucosyltransferase; Gal, galactose; GAPDH, glyceraldehyde-3-phosphate dehydrogenase; GlcNAc, N-acetylglucosamine; GnT, N-acetylglucosaminyltransferase; HPLC, high-performance liquid chromatography; Man, mannose; MDR, multidrug resistant protein; MIT, mitoxantrone; ODS, octadecylsilyl; PA, pyridylaminated; P-gp, P-glycoprotein.

## Competing interests

The author(s) declare that they have no competing interests.

## Authors' contributions

TK did mainly experiment whole of this paper.HN lectured oligosaccharide structural analysis and functions.

MT lectured mRNA expression and cell cultures.

JH helped TK experiment whole of this paper.NK established these drug-resistant cell lines.

HY lectured and helped cell culture.

KN organized mRNA expression experiments.

TN lectured and helped mRNA expressions.

TK lectured and helped mRNA expressions.

KD organized oligosaccharide structural analysis.

SIN organized oligosaccharide structural analysis.

ST organized whole of this paper.
